# Draft genomes of 16 *Bartonella henselae* strains from cats in Valdivia, Chile

**DOI:** 10.1128/MRA.00648-23

**Published:** 2023-10-17

**Authors:** Paulina Sepulveda, Marc Stegger, Gustavo Monti, Nivia Canales, Armin Mella, Patrick Butaye, Ananda Müller

**Affiliations:** 1 Escuela de Graduados, Facultad de Ciencias Veterinarias, Universidad Austral de Chile, Instituto de Medicina Preventiva Veterinaria, Valdivia, Chile; 2 Department of Bacteria, Parasites and Fungi, Statens Serum Institut, Copenhagen, Denmark; 3 Antimicrobial Resistance and Infectious Diseases Laboratory, Harry Butler Institute, Murdoch University, Perth, Australia; 4 Quantitative Veterinary Epidemiology, Wageningen University & Research, Wageningen, the Netherlands; 5 Instituto de Bioquímica y Microbiología, Universidad Austral de Chile, Valdivia, Chile; 6 Department of Biomedical Sciences, Ross University School of Veterinary Medicine, Basseterre, Saint Kitts and Nevis; 7 Department of Pathobiology, Pharmacology and Zoological Medicine, Ghent University, Ghent, Belgium; 8 Department of Infectious Diseases and Public Health, Jockey Club College of Veterinary Medicine and Life Sciences, City University of Hong Kong, Kowloon, Hong Kong; 9 Instituto de Ciencias Clínicas Veterinaria, Universidad Austral de Chile, Valdivia, Chile; University of Southern California, Los Angeles, California, USA

**Keywords:** *Bartonella henselae*, cat scratch disease, felids, bartonellosis, WGS

## Abstract

*Bartonella henselae* is a primary zoonotic agent, having cats as asymptomatic reservoirs. In humans, it causes cat scratch disease. Here, we report the whole genome sequences of 16 strains isolated from cats in Valdivia city, Southern Chile. Strains showed little variability in the multilocus sequence typing profiles.

## ANNOUNCEMENT

*Bartonella henselae* is a Gram-negative facultative intracellular bacterium (1), a primary zoonotic agent harbored by cats. The transmission between cats occurs via *Ctenocephalides felis* flea bites, while scratches and bites from cats are related to the zoonotic transmission. In humans, it causes cat scratch disease, while in cats it is usually asymptomatic (2, 3)

*Bartonella henselae* is fastidious to isolate (4) and only 38 whole genome sequences are available in the NCBI database (accessed 3 May 2023). In this study, 16 *B. henselae* strains from cat (from different households in Valdivia, Chile, 39.8142° S 73.2459° O) blood samples, collected by cephalic venipuncture between 2020 and 2021 (IACUC approval 353/2019), were isolated and submitted to whole genome sequencing.

For the culture and isolation, 100 µL of blood from 79 *Bartonella nuoG*-qPCR positive cats (5) was mixed with 100 µL Schneider’s Drosophila medium 1× (Gibco, Paisley, Escosia) and 5 µL of amphotericin B. This suspension was plated on chocolate agar medium and incubated at 37°C, 10% CO_2_ at high humidity for up to 60 days (4). Colonies suggestive of *Bartonella* spp. were confirmed at the genus level through *Bartonella nuoG*-qPCR (6). Sixteen pure isolates were obtained. DNA was extracted from single colonies scraped from the chocolate agar and purified using E.Z.N.A. Tissue DNA Kit (E.Z.N.A. Omega BioTek, USA). For *Bartonella* species identification, pure strain DNA was subjected to amplification and subsequent sequencing of the *gltA* gene (7).

DNA purity and concentration were determined using the Nanodrop (Thermo Scientific, Waltham, MA, USA) and Qubit (Invitrogen, Carlsbad, USA) instruments, respectively. Sequencing library preparation was accomplished using the Nextera XT DNA Library Preparation Kit (Illumina, San Diego, CA, USA) and sequenced on a MiSeq (Illumina, San Diego, CA, USA), using a paired-end 2 × 250 bp sequencing strategy, following standard Illumina protocols (Illumina, San Diego, CA, USA). The resulting sequences were quality controlled using Bifrost (https://github.com/ssi-dk/bifrost) to ensure adequate sequencing depth (minimum average of 25× coverage), species verification, and to identify contamination issues.

Raw sequences were trimmed and assembled using Unicycler version 0.4.8 (https://github.com/jmchandonia/kb_unicycler/tree/0a192937c92f1e6920306bb4d1bd437ced9f55c0/ui/narrative/methods/run_unicycler) and polished using Pilon version 1.23 as part of unicycler (module load Pilon/1.23-Java-11). Quality was assessed using Quast (version 5.0.2, fb0b821). The genome annotation was performed with the NCBI Prokaryotic Genome Annotation Pipeline version 6.5. All analyses were done on the BV-BRC server (https://www.bv-brc.org/). Multilocus sequence typing (MLST) profile was determined using *B. henselae* PubMLST (https://pubmlst.org/organisms/bartonella-henselae). Default parameters were used for all software unless otherwise specified.

The average size of the genomes was 1.82 Mbp with a minimum of 1.78 Mbp and a maximum of 1.84 Mbp. There were between 80 and 124 contigs. The *N*_50_ value was on average 54,604 bp. MLST profiles showed a relatively homogeneous collection of strains with mainly ST5 and only one ST1 and one ST6 (Table 1).

**TABLE 1:** Genome statistics and features of the *Bartonella henselae* strains isolated from cat blood^*a*^

Strain ID	No. of contigs	Total length (Mbp)	*N*_50_ (bp)	Genome coverage (×)	No. of reads	GC content (%)	Genbank accession no.	SRA accession no.	MLST strain type
BH13_S85	122	1,843,101	55,769	149.278	1,044,813	38.12	JAUDYR000000000	SRR18350077	1
BH16_S78	124	1,849,207	44,116	150.323	1,074,190	38.11	JAUDYT000000000	SRR18350070	5
BH1_S76	103	1,845,488	39,575	192.208	1,457,641	38.09	JAUDYP000000000	SRR18350078	5
BH20_S87	115	1,845,876	54,098	150.566	1,067,655	38.07	JAUDZB000000000	SRR18350069	5
BH25_S74	107	1,795,230	55,796	146.026	1,035,437	37.99	JAUDZC000000000	SRR18350068	5
BH27_S80	111	1,825,979	67,926	142.085	980,211	38.05	JAUDYX000000000	SRR18350067	5
BH34_S82	156	1,815,751	33,464	168.115	1,312,600	38.16	JAUDYZ000000000	SRR18350066	5
BH38_S84	124	1,829,502	51,099	153.039	1,078,299	38.05	JAUDZD000000000	SRR18350065	5
BH40_S86	115	1,844,064	63,720	111.580	921,061	37.96	JAUDYY000000000	SRR18350064	5
BH45_S88	80	1,780,245	67,579	155.827	1,207,302	37.93	JAUDZA000000000	SRR18350063	6
BH52_S91	114	1,826,852	57,210	130.003	925,390	38.06	JAUDYY000000000	SRR18350076	5
BH54_S92	106	1,834,120	61,133	178.019	1,357,367	38.04	JAUDYQ000000000	SRR18350075	5
BH56_S90	118	1,833,787	51,685	178.560	1,272,901	38.06	JAUDYV000000000	SRR18350074	5
BH58_S89	114	1,825,276	51,231	185.933	1,424,369	38.07	JAUDYS000000000	SRR18350073	5
BH61_S93	115	1,809,394	52,216	184.314	1,359,069	38.06	JAUDYU000000000	SRR18350072	5
BH63_S94	110	1,821,890	67,055	167.445	1,214,810	38.01	JAUDYW000000000	SRR18350071	5

^*a*^ MLST, Multilocus sequence typing.

## Data Availability

The whole-genome sequencing data and raw read data are available under BioProject accession number PRJNA817412. Accession numbers for the sequence reads and draft genomes are in Table 1.
